# PPARs and Tumor Microenvironment: The Emerging Roles of the Metabolic Master Regulators in Tumor Stromal–Epithelial Crosstalk and Carcinogenesis

**DOI:** 10.3390/cancers13092153

**Published:** 2021-04-29

**Authors:** Hong Sheng Cheng, Yun Sheng Yip, Eldeen Kai Yi Lim, Walter Wahli, Nguan Soon Tan

**Affiliations:** 1 Lee Kong Chian School of Medicine, Nanyang Technological University Singapore, 11 Mandalay Road, Singapore 308232, Singapore; ysyip@ntu.edu.sg (Y.S.Y.); walter.wahli@ntu.edu.sg (W.W.); 2 School of Biological Sciences, Nanyang Technological University Singapore, 60 Nanyang Drive, Singapore 637551, Singapore; LI0027YI@e.ntu.edu.sg; 3 Toxalim (Research Center in Food Toxicology), INRAE, ENVT, INP-PURPAN, UMR 1331, UPS, Université de Toulouse, 31300 Toulouse, France; 4 Center for Integrative Genomics, Université de Lausanne, Le Génopode, CH-1015 Lausanne, Switzerland

**Keywords:** peroxisome proliferation-activated receptor, metabolic reprogramming, cancer-associated fibroblast, cancer-associated adipocyte, tumor-associated macrophage

## Abstract

**Simple Summary:**

The roles of peroxisome proliferator-activated receptors (PPARs) in carcinogenesis are increasingly appreciated. With the growing interest in tumor stromal-epithelial crosstalk, we aim to provide an up-to-date overview of the implications of PPARs in the tumor microenvironment. In the tumor stromal cells, the nuclear receptors exhibit critical, but functionally diverse activities, rendering it hard to ascribe either an exclusive pro- or anti-tumorigenic role for different PPAR isotypes. Based on the existing evidence, we also highlight the knowledge gaps and future prospects of targeting PPARs in the tumor microenvironment. Essentially, a PPAR-based anticancer approach holds a great deal of untapped potential, but its success relies on innovative strategies for cell-specific or tumor microenvironment-triggered drug delivery systems.

**Abstract:**

Peroxisome proliferator-activated receptors (PPARs) have been extensively studied for more than three decades. Consisting of three isotypes, PPARα, γ, and β/δ, these nuclear receptors are regarded as the master metabolic regulators which govern many aspects of the body energy homeostasis and cell fate. Their roles in malignancy are also increasingly recognized. With the growing interest in crosstalk between tumor stroma and epithelium, this review aims to highlight the current knowledge on the implications of PPARs in the tumor microenvironment. PPARγ plays a crucial role in the metabolic reprogramming of cancer-associated fibroblasts and adipocytes, coercing the two stromal cells to become substrate donors for cancer growth. Fibroblast PPARβ/δ can modify the risk of tumor initiation and cancer susceptibility. In endothelial cells, PPARβ/δ and PPARα are pro- and anti-angiogenic, respectively. Although the angiogenic role of PPARγ remains ambiguous, it is a crucial regulator in autocrine and paracrine signaling of cancer-associated fibroblasts and tumor-associated macrophages/immune cells. Of note, angiopoietin-like 4 (ANGPTL4), a secretory protein encoded by a target gene of PPARs, triggers critical oncogenic processes such as inflammatory signaling, extracellular matrix derangement, anoikis resistance and metastasis, making it a potential drug target for cancer treatment. To conclude, PPARs in the tumor microenvironment exhibit oncogenic activities which are highly controversial and dependent on many factors such as stromal cell types, cancer types, and oncogenesis stages. Thus, the success of PPAR-based anticancer treatment potentially relies on innovative strategies to modulate PPAR activity in a cell type-specific manner.

## 1. Introduction

The year 2020 marks the 30-year discovery of nuclear hormone receptor, peroxisome proliferator-activated receptors (PPARs). In 1990, the first isotype of PPAR, now called PPARα, was successfully cloned from the mouse liver and identified as a novel nuclear receptor that is essential for triglyceride and cholesterol homeostasis [1]. Two years later, all three PPAR isotypes, namely PPARα, PPARβ/δ, and PPARγ, were isolated from the *Xenopus laevis* ovary and liver [2]. The research on PPARs has expanded exponentially ever since. Compelling evidence supports their roles as master regulators in metabolism and body energy homeostasis [3]. The clinical significance of PPARs is underscored by their synthetic ligands which are used to treat different facets of metabolic syndrome. Even before the discovery of PPARs, fibrates, which are PPARα agonists, have been used as lipid-lowering drugs and continue to be a mainstream therapy for atherogenic dyslipidemia and atherosclerosis [4]. Major synthetic PPARγ agonists, the thiazolidinediones (TZDs), are potent glucose-lowering agents that improve insulin sensitivity in adipose tissues and skeletal muscles [5]. To date, no PPARβ/δ ligand has been approved for clinical use. The clinical successes of TZDs and fibrates have spurred extensive development of next-generation PPAR ligands (i.e., antagonist, dual- and pan-PPAR agonists) for various metabolic complications, ranging from pre-morbid conditions such as obesity to chronic morbidities such as non-alcoholic fatty liver disease and chronic kidney disease [6]. Clearly, the discovery of PPARs underscores an important milestone in medicine, given the profound and pervasive impacts of PPARs in the way we tackle modern metabolic diseases.

The clinical impact of PPARs extends beyond metabolic disorders. To date, PPAR agonists have been trialed in many human diseases, including neurodegenerative disorders, psychiatric disorders, autoimmune and inflammatory diseases, as well as malignancies, with varying degrees of success [6,7]. PPAR-related metabolic dysregulations, such as obesity and type 2 diabetes, are independent risk factors of carcinogenesis and cancer prognosis predictors [8,9]. Thus, there is intense research spotlight on exploiting PPARs for cancer therapy. Early investigations revealed that, in the majority of cases, the activation of PPARβ/δ is linked to tumor progression, whereas PPARα and PPARγ are associated with anti-tumorigenesis [10]. Nevertheless, existing cancer trials revealed a huge cancer-to-cancer discrepancy, undermining the potential of PPAR ligands in cancer therapy [6]. Such discordance between preclinical and clinical outcomes indicates unaccounted hidden players interacting with PPARs during carcinogenesis.

It is now well-recognized that cancer cells do not live in a rigid and homogenous mass, but rather in a highly dynamic and heterogeneous community comprising a wide variety of cell types such as fibroblasts, adipocytes, immune cells, endothelial cells, pericytes, and mesenchymal stem cells, collectively known as the tumor stromal cells [11]. The interplay between tumor stromal cells and the epithelium is crucial to every step of tumorigenesis, from initiation, progression, and metastasis, besides offering enhanced plasticity and resistance to various stressors and physiological cues in cancer cells [12]. Increasing evidence also implicates a profound role for PPARs in stromal cellular behaviors and eventual consequences in cancer hallmarks. Our review aims to consolidate the current understanding of PPAR-mediated activities in carcinogenesis and tumor stromal–epithelial communication.

## 2. The Roles of PPARs in Tumor Epithelium

### 2.1. Functional Diversity of PPARs in Tumorigenesis

#### 2.1.1. PPARα

**Figure 1:**
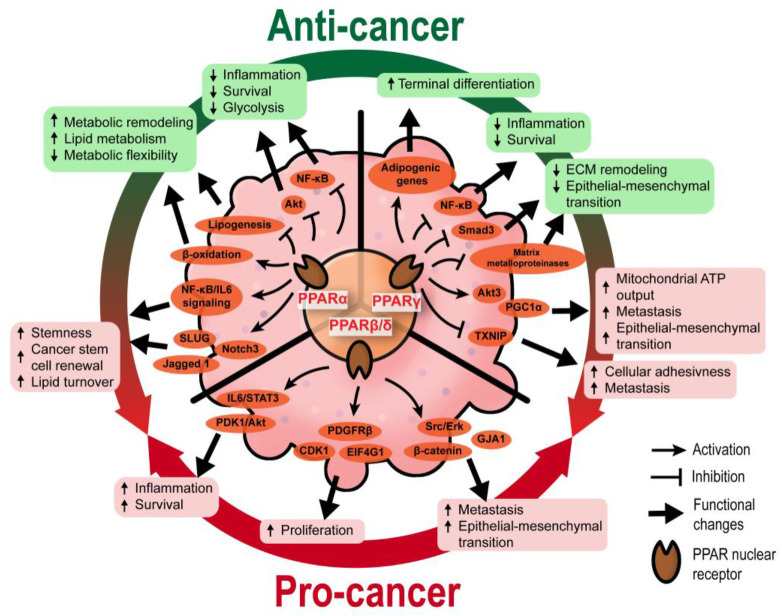
The cellular activities regulated by PPARs in tumor epithelium. In a tumor cell, PPARα and PPARγ exhibit controversial roles. They are generally linked to anticancer effects (green text boxes) by impairing the pro-inflammatory, pro-metastatic, and pro-survival responses, as well as reducing metabolic flexibility. However, their pro-cancer activities (red text boxes), including the maintenance of cancer stemness, meeting high energy demands of cancers and promoting metastasis, have been reported. On the other hand, PPARβ/δ activates signaling pathways and key mediators implicated in pro-cancer activities such as enhanced survival, proliferation, and epithelial–mesenchymal transition. ECM, extracellular matrix.

The three PPAR isotypes have diverse physiological functions and expression patterns in different tissues. Likewise, they also possess vastly different roles in cancer cells (Figure 1). Marked species differences are apparent in response to peroxisome proliferation induced by activated PPARα. Rats and mice are extremely sensitive, while humans appear to be relatively insensitive or non-responsive at dose levels that produce a marked carcinogenic response in rodents. Experimental evidence suggests a probable link between peroxisome-proliferator-elicited liver growth and the subsequent development of liver tumors in rats and mice. In rodents, the activation of PPARα induces miRNA-mediated neoplastic changes in the liver [13]. However, these oncogenic events are not recapitulated in PPARα humanized mice and human hepatocyte cell lines [14].

A few studies also reported a pro-carcinogenic role of PPARα. In a small-scale cross sectional study (*n* = 100 patients), the overexpression of PPARα in the tumor microenvironment (TME) of colorectal cancer has been linked to poorer prognosis [15]. In breast cancer stem cells, GW6471 (a PPARα antagonist) is anti-proliferative and pro-apoptotic, while Wy14643 (a PPARα agonist) induces the clonal expansion of breast cancer mammospheres by promoting the signaling activities of the nuclear receptor κB (NF-κB)/ interleukin-6 (IL-6) axis, SLUG, Notch3, and Jagged 1 [16,17]. PPARα signaling also ensures a high lipid turnover rate, sustaining the high energy demand to maintain stemness and self-renewal in pancreatic and colorectal cancer stem cells [18].

Based on a meta-analysis, the clinical use of fibrates, which can be traced back to the mid-1970s, does not significantly increase cancer incidence [19]. In fact, PPARα activities are primarily thought to be anticancer in humans. The nuclear receptor can repress the oncogenic roles of NF-κB and Akt, besides forcing the tumor cells to adopt a lipo-centric metabolism [20,21]. Consequently, the tumor cells which adapt poorly to the PPARα-mediated anti-inflammatory response and enhanced fatty acid oxidation may become less proliferative and undergo apoptotic, necrotic, or autophagic cell death.

#### 2.1.2. PPARγ

Most studies support an anti-carcinogenic role for PPARγ, as summarized in a recent review [22]. A high expression of PPARγ is associated with a favorable prognosis in colorectal cancer patients [15]. The activation of PPARγ in cancer cells stimulates adipogenesis and disrupts the Hippo-YAP signaling pathway to force terminal differentiation and suppress proliferation [23,24,25]. Many cancer stem cells are also sensitive to the terminal differentiation directed by PPARγ [24,26,27]. PPARγ-mediated *PTEN* upregulation inhibits PI3K signaling to diminish the self-renewal and aggressiveness of cancer stem cells [28,29]. Furthermore, PPARγ agonists trigger NF-κB transrepression and modulate various BCL-2 family proteins such as BAX, BAD, Bcl-XL, Bcl2, and PI3K/Akt c-Jun to exert anti-inflammatory and pro-apoptotic properties [21]. PPARγ agonists, ciglitazone and 15-deoxy-Δ12,14-prostaglandin J2 (15d-PGJ2), inhibited cell viability and proliferation of brain tumor stem cells at least via the inhibition of Sox2 while enhancing Nanog expression [30]. The differential regulation of Sox2 and Nanog by PPARγ agonists suggests a critical role for these stemness factors in modulating the growth and differentiation of stem cells in glioma. However, the mechanism by which PPARγ agonists regulate differentiation and self-renewal remains unclear. Separately, by suppressing matrix metalloproteinases and antagonizing Smad3-dependent transcriptional activity, PPARγ also attenuates extracellular matrix (ECM) remodeling and epithelial–mesenchymal transition (EMT), which, in turn, leads to reduced tumor metastasis [31,32].

Reports on the pro-cancer effect of PPARγ are not uncommon. Yang et al. (2005) [33] and Pino et al. (2004) [34] concluded that the use of PPARγ agonists is associated with increased cancer incidence in genetic mice models of colorectal cancer. A few studies have also described the increased risk of PPARγ agonists for renal and bladder cancers [35,36]. Several molecular mechanisms for the pro-cancer effect of PPARγ have been proposed. For instance, Galbraith et al. (2021) [37] demonstrated that in prostate cancer, PPARγ overexpression promoted the activity of Akt3, which subsequently inhibited a nuclear export protein, CRM1, and enhanced the nuclear retention of PPARγ co-activator 1α (PGC1α). Such activity ramps up the mitochondrial ATP output in cancer cells to meet the exorbitant energy demand for EMT and metastasis. In human melanoma, the activation of PPARγ remodels the expression and localization of surface integrins, particularly integrin β-3 and integrin α-5, to increase cellular adhesiveness and distal metastatic seeding [38]. These metastatic phenotypes are linked to the suppression of thioredoxin-interacting protein (*TXNIP*), whose expression is negatively regulated by PPARγ [38]. Moreover, using a liver-specific *Pten* knockout mouse model, it was found that Akt2 promotes the activation and pro-tumorigenic signaling of PPARγ by repressing hepatocyte nuclear factor 1α (HNF1α) [39,40]. Although multiple pro-tumorigenic mechanisms of PPARγ have been found, to date, there is no consensus if these PPARγ-mediated pathways are ubiquitous in different cancer cell types. Notably, the genetic background could act as a strong modifier of the pro-tumor effect of PPARγ, as examplified by the predisposition of certain PPARγ polymorphisms (i.e., Pro12Ala and C161T) to breast cancer [41]. The genetic predisposition would also explain why certain ethnic groups may be more susceptible to the cancer onset with prolonged usage of PPARγ agonists even though TZDs are generally associated with protective effects against several common cancers [42,43].

#### 2.1.3. PPARβ/δ

The dual role of PPARβ/δ in cancer has been thoroughly reviewed [44,45]. In summary, most of the studies are in favor of a pro-tumorigenic profile of PPARβ/δ. Extensive investigations were focused on colon cancers [15]. Pro-tumorigenic activities of PPARβ/δ have been demonstrated in many colon cancer mouse models, including Apc^Min/+^ mice [46], azoxymethane-induced colon tumors [47], colitis-associated colon cancer [48], high-fat diet or PPARβ/δ agonist-treated mice [49]. PPARβ/δ overexpression exacerbates the activation of β-catenin and several pro-invasive pathways, namely connexin 43, PDGFRβ, Akt1, EIF4G1, and CDK1, to promote colorectal cancer progression [46]. PPARβ/δ also positively regulates IL-6/STAT3-mediated inflammation and many pro-metastatic genes [48,50]. PPARβ/δ is a key mediator of PDK1-mediated mammary carcinogenesis [51]. In a nonmelanoma skin cancer mouse model, PPARβ/δ activates the oncogene Src and the EGFR/Erk1/2 signaling pathways upon UV exposure, resulting in increased tumor burden and EMT [52]. Enhanced response of Erk to transforming growth factor β1 (TGF-β1) is also seen in prostate cancer cells, in response to PPARβ/δ-mediated activation of ABCA1 and caveolin-1, which results in TGF-β1-induced tumor growth, migration, and invasion [53]. In terms of cancer stem cells, the current understanding of the role of PPARβ/δ is somewhat lacking. A recent study revealed that PPARβ/δ upregulates Nanog expression in colorectal cancer cells, promoting metastasis when exposed to a fat-enriched environment [54]; yet, another study showed its suppressive effect on SOX2 expression, thus inhibiting neuroblastoma tumorigenesis [55]. While the pro-tumorigenic role of PPARβ/δ in tumor epithelium is well-supported, opposite findings have also been reported [56,57,58]. The conflicting results suggest other still hidden mechanisms that can fine-tune the cellular activity of PPARβ/δ towards pro- or anticancer effects.

### 2.2. Clinical Development of PPAR Modulators as Cancer Therapeutics

The tight entanglement of PPAR signaling and tumorigenesis leads to the repurposing of PPAR-targeting drugs for cancer treatment. Many early phase clinical trials have been conducted to examine the clinical feasibility of PPAR agonists, particularly PPARα and PPARγ agonists, against a wide range of cancers [6]. However, existing evidence does not support using any PPAR modulators to treat cancers because of underpowered study design, marginal effect size, and underwhelming outcomes. The discrepancy between preclinical and clinical results highlights a knowledge gap in our understanding of PPARs in carcinogenesis. In fact, PPAR activities may vary across different cancer types and stages. On top of that, the TME adds an extra layer of complexity to the regulatory roles of PPARs in oncogenic processes, which existing PPAR cancer research often fails to take into consideration. As PPARs may have vastly distinct roles in tumor stromal cells compared to epithelial cells during tumorigenesis, in the next section, we will provide an overview of the current understanding of PPARs in the TME and the interplay between tumor stroma and epithelium.

## 3. The Roles of PPARs in Stromal Cells in the Tumor Microenvironment

Most anticancer therapies target malignant cancer cells while largely ignoring the surrounding noncancer cell components of the tumor or TME. The TME or tumor stroma comprises nonmalignant host cellular and acellular components, including, but not limited to, fibroblasts, immune cells, endothelial cells, fat cells, and noncellular components of the tumor niche such as the basement membrane and ECM. Although most normal host cells in the stroma possess certain tumor-suppressing abilities, the stroma will change during malignancy, causing the tumor stromal cells to confer pro- or anti-tumor properties in a context- and cell type-dependent manner. Over the past decades, the role of the TME in determining every aspect of cancer progression and the efficacy of treatment has become evident. The functions of PPARs in these stromal cells are increasingly appreciated and have direct or indirect impacts on cancer progression.

### 3.1. PPARγ: A Master Regulator of Stromal Metabolic Reprogramming

#### 3.1.1. Cancer-Associated Fibroblasts

Cancer metabolism and bioenergetics are vastly different from those of normal epithelial cells. A high basal metabolic rate, coupled with abnormal vasculatures in the TME, poses a tremendous challenge for cancer cells to fulfill their energy demand. While the cancer cells possess remarkable plasticity and versatility to utilize various substrates to meet their demand for cellular energy, the surrounding stromal cells also play an indispensable role during cancer progression.

Under the paracrine influences of cancer cells, stromal cells such as cancer-associated fibroblasts (CAFs) and cancer-associated adipocytes (CAAs) can transform into substrate donors to provide fuels and building blocks, namely glutamine, L-lactate, fatty acids, and ketone bodies. These metabolites are readily channeled into the Krebs cycle and oxidative phosphorylation of the cancer cells for ATP generation [59,60]. PPARγ governs many processes involved in the metabolic remodeling of stromal cells. Clinically, the expression of PPARγ is significantly upregulated in CAFs of cutaneous skin squamous cell carcinoma and colon adenocarcinoma [61,62]. In one study, immortalized human fibroblasts overexpressing PPARγ were more glycolytic, autophagic, and displayed a senescent phenotype [63]. L-lactate secretion also increased by 70% in PPARγ-overexpressing fibroblasts compared to wild-type counterparts [63]. These PPARγ-induced metabolic features are typical in a tumor-supporting stroma, as evidenced by accelerated tumor xenograft growth of MDA-MD-231 breast cancer cells when co-implanted with transgenic fibroblasts overexpressing PPARγ, but not with wild-type fibroblasts [63].

The hypoxic TME further aggravates the autophagic phenotype in tumor stromal cells, suggesting a modifying role of hypoxia-inducible factor 1α (HIF-1α) in PPARγ-dependent autophagy [63,64]. Furthermore, a study on a genetic defect (*MTO1* deficiency) in mitochondria reported that AMP-activated protein kinase (AMPK) and uncoupling protein 2 (UCP2) interacted closely with PPARγ and HIF-1α, generating a HIF1α-PPARγ-UCP2-AMPK axis, to influence mitochondrial bioenergetics and key metabolic processes such as glycolysis, fatty acid oxidation, and oxidative phosphorylation, leading to extensive metabolic reprogramming in fibroblasts [65]. AMPK ensures the maturation of autophagosome and lysosomal fusion during autophagy [66], besides modulating the genes responsible for mitochondrial integrity (*UCP2* and *PGC-1α*), autophagy (*BECN-1*, *LC3B*, *ATG5*, *ATG7*, and *SQSTM1*), and mitophagy (*PINK1*, *FUNDC1*, *BNIP3*, and *PRKN*) [67]. The expression of AMPK target genes is considerably disrupted in fibroblasts overexpressing PPARγ under normoxia and hypoxia [63]. As such, the interplay among PPARγ, HIF1α, and AMPK is pivotal in modulating CAF autophagy, but the exact mode of interaction remains largely elusive.

Following autophagy, glycolysis occurs to recycle cellular organelles and debris into basic building blocks reusable by cancer cells [68,69]. Many glycolytic genes are subject to PPARγ regulation [70,71]. Several studies also pointed to NF-κB as a key transcription factor of stromal autophagy and glycolysis [63,72], but its interaction with PPARγ remains elusive. In short, PPARγ regulates key genes and cellular events in CAFs to accomplish the metabolic coupling of tumor stroma and epithelium, essentially transforming CAFs into a powerhouse that constantly generates energetic biomolecules to support tumor growth.

In contrast to the tumor-supporting properties of CAFs overexpressing PPARγ, pharmacologic PPARγ activation in tumor epithelium confers anticancer effects by reducing tumor proliferation and neovascularization [63]. Thus, the activation of PPARγ metabolically reprograms CAFs to favor autophagic and glycolytic behaviors, allowing cancer cells to use nutrients from non-autonomous sources to sustain their uncontrolled proliferation and other activities.

#### 3.1.2. Cancer-Associated Adipocytes

Like CAFs, CAAs also serve as storage sites and nutrient donors in the TME [73]. Fibroblasts and mesenchymal stromal cells readily undergo adipogenesis and differentiate into adipocytes upon exposure to adipogenic stimuli, especially the activation and upregulation of PPARγ [74,75]. Cancer exosomes loaded with miRNA-144 and miRNA-155 facilitate the beige/brown differentiation of CAAs by modulating the MAP3K8-Erk1/2-PPARγ axis, whereas those carrying miRNA-126 can disrupt IRS-GLUT4 signaling and promote AMPK- and HIF1α-mediated autophagy [76,77]. Cancer cells can also initiate the dedifferentiation of adjacent adipocytes, a process that is consistently observed when adipocytes are cocultured with cancer cells [78,79]. The process is characterized by the progressive loss of mature adipocyte markers such as leptin, adiponectin, HSL, and PPARγ, increased expression of fibroblast markers such as matrix metalloproteinase 11 (MMP11), collagen I, and α-SMA, as well as the adoption of a fibroblast-like morphology in the cocultured adipocytes [78,79]. These dedifferentiated adipocytes exhibit transcriptional suppression of *GLUT4* and *IRS1* and inhibit insulin-induced Akt phosphorylation [78]. These aberrations occur alongside the downregulation of MAP3K8-Erk1/2-PPARγ, effectively escalating the catabolic capacity of CAAs to secrete pyruvate, L-lactate, and ketone bodies [76].

Moreover, diminished ligand activation of PPARγ through the constitutive expression of Notch1 induces adipocyte de-differentiation and tumor-like manifestations [80]. Treatment with rosiglitazone, a PPARγ agonist, effectively promoted adipocyte redifferentiation and attenuated the transformation of the adipocytes [80]. Consistent with these observations, the adipocyte-specific deletion of *PPARγ* in a chemically induced breast cancer model impaired *BRCA1* expression in CAAs and subsequently accelerated tumor formation and progression [81]. Undoubtedly, PPARγ is a critical mediator in the cellular fate and metabolic reprogramming of CAAs. Although the actual functionality of adipocyte dedifferentiation in tumor stroma remains unclear, it is generally associated with pro-tumorigenic activities [76,78]. Furthermore, dedifferentiated adipocytes can be redifferentiated into other cell lineages, including beige/brown adipocytes that readily release bioenergetic molecules into the TME [82]. Such plasticity of adipocytes entails the possibility for tumor cells to coerce the CAAs into other tumor supportive cells.

**Figure 2:**
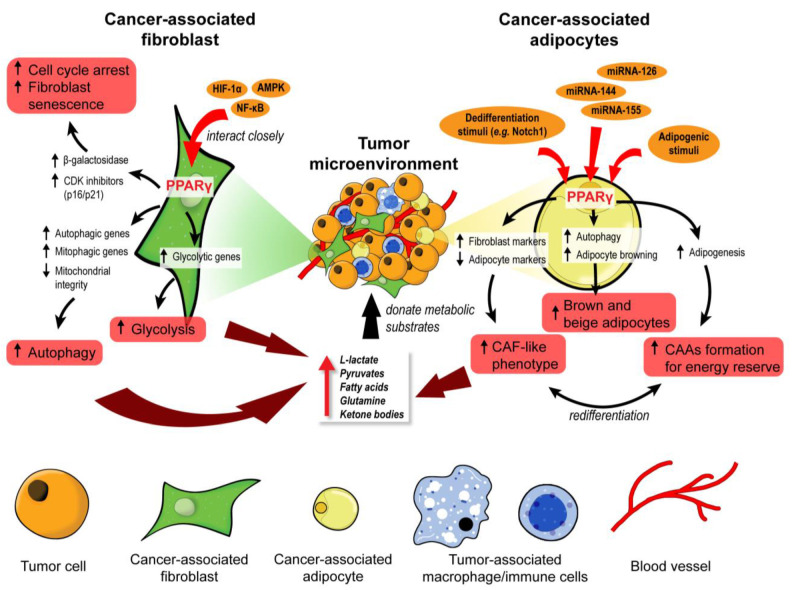
PPARγ orchestrates the metabolic reprogramming of cancer-associated fibroblasts and adipocytes. In cancer-associated fibroblasts (CAFs), PPARγ interacts closely with HIF-1α, AMPK, and NF-κB to promote cell cycle arrest, senescence, autophagy, and glycolysis. These functional changes unleash many metabolic substrates into the tumor microenvironment for the neighboring tumor cells. Similarly, PPARγ governs the fate and function of cancer-associated adipocytes (CAAs). Upon exposure to adipogenic stimuli, PPARγ mediates adipogenesis and formation of CAAs to act as an energy reserve. In contrast, exposure to dedifferentiation stimuli drives CAAs to adopt a CAF-like phenotype and act as a substrate doner in the tumor microenvironment. Certain miRNAs can suppress PPARγ to induce brown and beige differentiation of CAAs which are also energy donors for cancer progression.

Taken together, CAFs and CAAs are two key stromal cells that undergo extensive metabolic reprogramming to act as energy reserves for cancer epithelium, as illustrated in Figure 2. PPARγ signaling is implicated in the remodeling of both stromal cells, but the activity is vastly different. Autophagic CAFs are triggered by PPARγ activation, while PPARγ is suppressed in dedifferentiated CAAs. This cell type-dependent disparity highlights a need for strategies to target PPARγ in a cell-specific manner so that the treatment is not counter-productive.

### 3.2. PPARβ/δ in CAFs Governs Redox Homeostasis and Affects Tumor Initiation

The differentiation of normal fibroblasts into CAFs is one of the cornerstones of early tumor initiation in many cancer types [83,84]. CAFs can disrupt the local ECM and deliver proliferative paracrine signals to support tumorigenic events. Interestingly, mice with fibroblast-selective *PPARβ/δ* deletion developed fewer and smaller skin tumors than wild-type mice exposed to topical carcinogens [85]. Similar results were recapitulated using chemically and genetically induced intestinal carcinogenesis in these mutant mice [86], indicating that PPARβ/δ activity in stromal fibroblasts promotes tumor initiation. The delayed tumor emergence in the mutant mice was due to an enhanced antioxidant response in the epithelium. Mechanistically, PPARβ/δ-knockout fibroblasts markedly increase the Nox4-derived H_2_O_2_ production in the adjacent epidermis, subsequently triggering an RAF/MEK-mediated NRF2 activation that elicits a strong antioxidant and cytoprotective response [85]. By reducing the phosphorylation of many tumor suppressors and oncogenes, NRF2 also increases the tumor suppressor activity of PTEN and reduces the oncogenic activity of Src and Akt, leading to delayed tumor growth [85]. Hence, reducing the expression and activity of *PPARβ/δ* in CAFs may provide a new therapeutic option to disrupt cancer susceptibility in the neighboring tumor epidermis.

Leucine-rich-alpha-2-glycoprotein 1 (LRG1) and TGFβ1 underpin a crucial process in the PPARβ/δ-mediated stromal–epithelial crosstalk. PPARβ/δ in fibroblasts upregulates the expression of LRG1, which blunts the epidermal response to TGFβ1 [87]. Furthermore, exogenous LRG1 can also ablate the influence of TGFβ1 on ROS generation and NRF2 activity [85]. In colorectal carcinoma and pancreatic ductal adenocarcinoma patients, the level of LRG1 in the TME and bloodstream is significantly higher than in healthy individuals and correlates positively with a more advanced cancer stage and poorer prognosis [88,89,90]. This observation suggests a pro-tumorigenic role of LRG1. Surprisingly, the *LRG1* promoter has two putative PPAR response elements [91]. The expression of *LRG1* is increased by a PPARβ/δ agonist, GW501516, which strongly suggests that LRG1 is a direct target of PPARβ/δ [91]. Therefore, during the early stage of tumorigenesis, CAF PPARβ/δ may stimulate LRG1 expression, which interferes with TGFβ1-dependent redox homeostasis, to support a sustained oncogenic transformation in the surrounding tumor epithelium.

**Figure 3:**
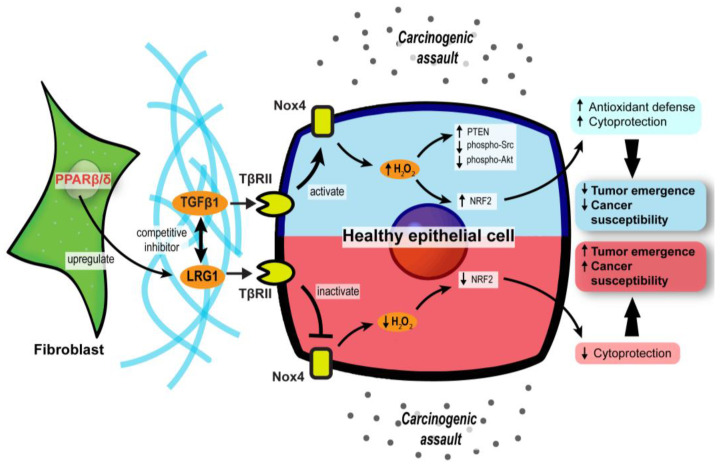
Stromal PPARβ/δ regulates epithelial redox homeostasis and oncogenesis. In carcinogenic assaults, TGFβ signaling in epithelial cells is activated to promote H_2_O_2_ synthesis, which subsequently activates NRF2 and reinforces the cytoprotection against carcinogens (blue upper compartment of the epithelial cell). However, fibroblast PPARβ/δ disrupts the protective mechanism by upregulating LRG1, which acts as a competitive inhibitor of TGFβ1 and dampens TGFβ signaling, resulting in increased cancer susceptibility and oncogenesis (red lower compartment of the epithelial cell).

Collectively, these findings uncover a major role for stromal PPARβ/δ in the epithelial–mesenchymal communication and cellular oxidative response in tumor development (Figure 3). Notably, this novel role of PPARβ/δ was primarily documented, so far, in nonmelanoma skin carcinoma and colorectal cancer models. Thus, further validation in other cancer models is necessary.

### 3.3. Endothelial PPARs Affect Angiogenesis in the Tumor Microenvironment

Hypoxic regions often arise because of rapid tumor growth, which outgrows the oxygen perfusion and nutrient supply from existing vasculature [92]. Cancer cells mitigate the predicament by releasing pro-angiogenic factors that stimulate angiogenesis, which is affected by all three PPAR isotypes.

In terms of PPARα, synthetic PPARα agonists such as fenofibrate and Wy-14643 have demonstrated suppressive effects on endothelial cell proliferation, neovascularization, and tumor xenograft growth [93,94]. Such anti-angiogenic effects of PPARα agonists were lost in PPARα-deficient mice transplanted with PPARα-intact tumor cells, implying that PPARα activation in surrounding stromal cells, but not the tumor cells, attenuated tumor angiogenesis [93,94]. The underlying mechanism is associated with increased anti-angiogenic factors (i.e., thrombospondin-1 and endostatin) and the interference of pro-angiogenic factor biosynthesis (i.e., VEGF-A, angiopoietin-1, and angiopoietin-2), affecting VEGF- and FGF2-mediated endothelial proliferation and migration [93,95]. Furthermore, by transcriptionally suppressing the expression of endothelial P450 CYP2C epoxygenase, whose function is to catalyze arachidonic acid epoxidation, PPARα also diminishes the epoxygenase products, epoxyeicosatrienoic acids, which are pro-angiogenic [96]. Thus, PPARα activation in stromal endothelial cells inhibited the biosynthesis of pro-angiogenic factors while promoting the secretion of anti-angiogenic factors, thereby abrogating angiogenesis and limiting nutrient supply to attenuate tumor progression.

In contrast to PPARα, PPARβ/δ is a pro-angiogenic nuclear receptor in line with its wound healing properties [97,98,99]. The activation of PPARβ/δ in endothelial cells by synthetic ligands or genetic manipulation consistently results in aberrant biosynthesis of VEGF, PDGFR, and c-KI, as well as accelerated endothelial cell proliferation and vascular formation [100,101]. In the TME, these pro-angiogenic changes stimulate the formation of a tumor with a higher vessel density, enhancing tumor feeding, oxygen provision, and metastasis capacity of the cancer cells [101]. Interestingly, in PPARβ/δ knockout mice harboring experimental wild-type tumors, the endothelial cells forming the microvessels in the tumors appear immature, hyperplastic, and less well-organized, leading to abnormal microvasculature and restricted blood flow into the tumors [102,103]. Apart from conventional growth factors, other potential PPARβ/δ-dependent angiogenic mediators include CDKN1C [102], IL-8 [104], CLIC4, and CRBP1 [105]. Considering its regulatory effects on many angiogenic genes and the strong linkages with advanced cancer stages, tumor recurrence, and distant metastasis, PPARβ/δ is identified as one of the pro-angiogenic signaling hubs in cancers [103]. Thus, the pro-tumorigenic and pro-angiogenic activities of PPARβ/δ warrant the development of efficacious PPARβ/δ antagonists to be tested in cancer models.

Existing evidence on the role of PPARγ in angiogenesis remains ambiguous. Like PPARα, PPARγ activities in the TME are associated with the dysregulated production of angiogenic factors, especially platelet-derived endothelial cell growth factor (PD-ECGF) and fibroblast growth factor (FGF) [106,107]. Early studies generally concluded on an inhibitory effect of PPARγ ligands on endothelial cell proliferation in response to pro-angiogenic factors and endothelial tube formation [108,109], whereas subsequent investigations suggested otherwise [110,111]. Such conflicting findings may be attributable to the dosages of PPARγ ligands and endothelial cell types [112]. Regardless of the pro- or anti-angiogenic properties, VEGF/VEGFR signaling is coherently implicated in the PPARγ-mediated effect [108,109,110]. A recent study using endothelial-specific PPARγ knockout models shed new light on the role of this nuclear receptor in angiogenesis. In mature endothelial cells, PPARγ knockdown impaired proliferation, migratory properties, and tubule formation capacity [111]. These impairments translated into the loss of circulating endothelial progenitor cells and angiogenic capacity in endothelial-specific PPARγ-deficient mice, which was reversed by the transplantation of wild-type bone marrow [111]. Mechanistically, abolishing PPARγ in the endothelial cells disrupts E2F1-mediated Wnt signaling and GSK3B interacting protein activity, resulting in suppressed endothelial proliferation [111]. Conceivably, the genetic models reinforce the pro-angiogenic activity of PPARγ in endothelial cells.

**Figure 4:**
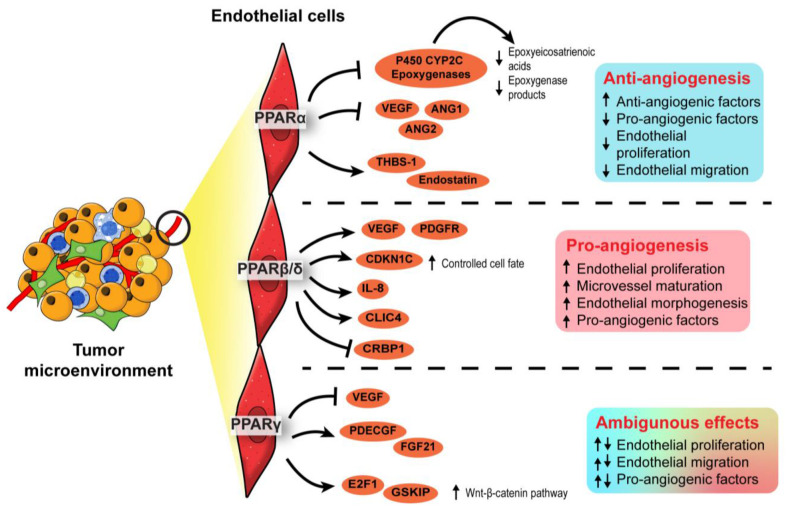
Angiogenic role of PPARs in endothelial cells. In the endothelial cells, PPARα exhibits an anti-angiogenic effect by inhibiting endothelial proliferation, whereas PPARβ/δ appears pro-angiogenic by ensuring proper endothelial morphogenesis and vascular maturation. The role of PPARγ in angiogenesis is conflicting and warrants further investigation.

In short, PPARα and PPARβ/δ exert anti- and pro-angiogenic activities in the endothelial cells of TME, respectively. On the other hand, opposing roles have been reported for PPARγ in angiogenesis. The roles of each PPAR isotype in angiogenesis are summarized in Figure 4. Notably, most findings on PPARγ are not established using oncogenic models. As the physiological cues in a TME are different from a normal condition, the true nature of PPARγ in cancer angiogenesis and tumor epithelium-endothelium crosstalk requires further investigation.

### 3.4. PPAR-Dependent Autocrine and Paracrine Signaling

**Figure 5:**
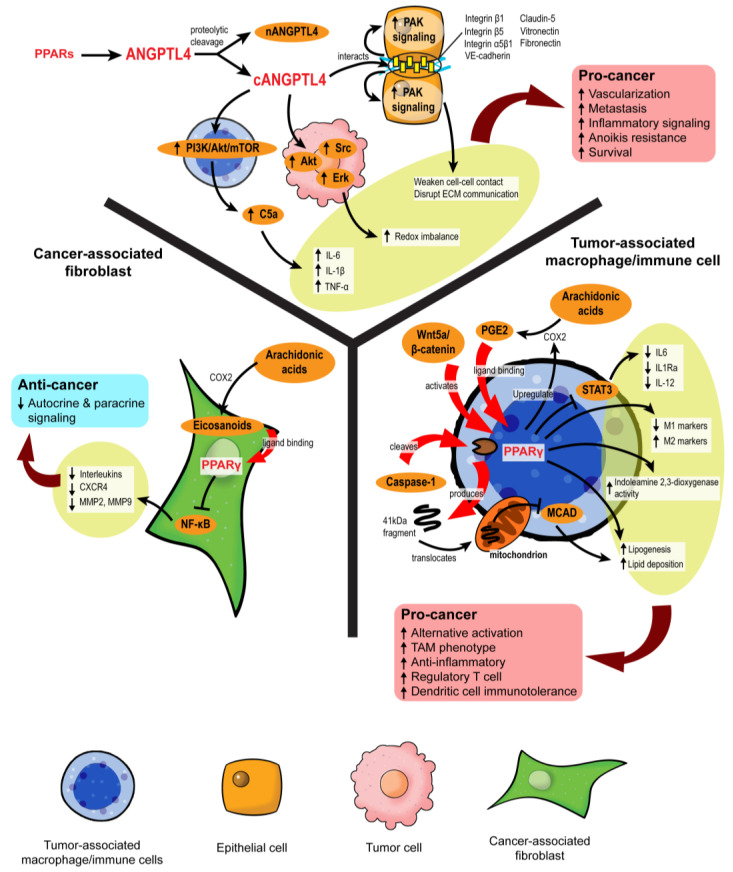
PPARs modulate stromal–epithelial crosstalk in the tumor microenvironment. PPARs affect autocrine and paracrine signaling in different stromal cells. In cancer-associated fibroblasts, PPARγ activation upon ligand binding represses NF-κB, alleviating the secretion of many autocrine and paracrine signals. However, in macrophages and immune cells, PPARγ activation is primarily linked to pro-cancer activities, such as the formation of tumor-associated macrophages (TAMs), increased regulatory T cells, and immunotolerance. ANGPTL4 is a target gene product of PPARs. Proteolytic cleavage of full-length ANGPTL4 yields nANGPTL4 and cANGPTL4 domains, of which the latter is a potent paracrine signal and key mediator of inflammatory signals, anoikis resistance, and metastasis.

Autocrine signaling facilities self-stimulation, while paracrine signaling allows local cell–cell communication. In the TME, both forms of cell signaling are imperative to coordinate every stage of oncogenesis, alerting the tumor cells how and when to proliferate, evade immune surveillance, escape from the existing microenvironment, and settle at a distal site. The transmission of complex messages in response to cellular stimuli is made possible by a plethora of secretory mediators, including cytokines, chemokines, growth factors, catalytic proteins, miRNAs, extracellular vesicles, and lipid compounds [113]. Many of these messengers are directly or indirectly regulated by PPARs (Figure 5). For instance, a new PPARγ agonist, CB13, remodels the exosomal contents from radio-resistant non-small cell lung cancer to promote endoplasmic reticulum stress and cell death via a PERK-eIF2α-ATF4-CHOP axis [114].

#### 3.4.1. Disruption of Pro-Tumor Signaling by PPARγ in CAFs

Eicosanoids, which are lipid signaling molecules and cognate ligands of PPARs, are the main drivers of PPAR activation in the TME. Major eicosanoid subfamilies include prostaglandins, thromboxanes, leukotrienes, and epoxygenated fatty acids, among which the prostaglandins are the most well-investigated. In colon cancers, cyclooxygenase-2 (COX-2), an enzyme that catalyzes the conversion of arachidonic acid to prostaglandin H2 (PGH2), is overexpressed in CAFs surrounding colon adenocarcinomas, leading to a buildup of intratumoral PGE2 [61,115]. However, the resultant activity of PPARs varies across different stromal cells. For instance, 15d-PGJ2 activates PPARγ and suppresses the proliferation of CAFs and expression of the ECM remodeling enzyme, MMP2 [116]. By inhibiting NF-κB, TZD-activated PPARγ substantially lowers the expression of pro-inflammatory, pro-angiogenic, and pro-metastatic signaling molecules in CAFs, including IL-6, IL-8, CXCR4, MMP2, and MMP9, which further dampens pro-tumor crosstalk in the TME [117,118]. The repression of PPARγ activity also disturbs the quiescent state of hepatic and pancreatic stellate cells, compelling their differentiation into CAFs with highly aggressive phenotypes and inducing desmoplasia in the TME [119,120,121,122]. Despite some conflicting results [123], PPARγ in CAFs can disrupt pro-tumorigenic paracrine signaling by suppressing the liberation of cytokines and chemokines.

#### 3.4.2. PPARγ Propels the Formation of Tumor-Associated Macrophages

The role of PPARs in innate and adaptive immune cells has been extensively studied. Unlike CAFs, the activation of PPARα and PPARγ in macrophages favors an anti-inflammatory tumor-associated macrophage (TAM) phenotype [124,125]. Classical PPARγ ligands, namely rosiglitazone, *N*-docosahexaenoyl ethanolamide, and *N*-docosahexaenoyl serotonin, effectively block paracrine signals from cancer cells to sway the fate of macrophages to adopt alternative activation and reduce their STAT3-mediated pro-inflammatory response [125]. In macrophages challenged with pathogens, WY14643 (PPARα agonist) and 15d-PGJ2 (PPARγ agonist) tip the balance towards the M2 phenotype by enhancing the expression of arginase I, Ym1 (chitinase 3-like 3), mannose receptor, TGF-β and increasing phagocytic capacity while diminishing M1 macrophage biomarkers [126]. PPARγ antagonists and macrophage-specific PPARγ ablation attenuate these effects, clearly outlining the dependency of TAM differentiation on PPARγ [127,128].

Mechanistically, PPARγ agonism promotes lipid retention, lipogenesis, and PGE2 secretion in macrophages. The lipid metabolic changes are partly mediated by the Akt/mTOR pathway [129]. On top of its role as a nuclear receptor and transcription factor, PPARγ is subject to cleavage by caspase-1 to yield a 41 kDa fragment that translocates to mitochondria and inhibits medium-chain acyl-CoA dehydrogenase (MCAD). Such a non-canonical peptide–protein interaction can inhibit fatty acid oxidation, further aggravating lipid droplet accumulation and TAM formation [130]. Likewise, in dendritic cells residing in the TME, PPARγ activation directed by Wnt5a/β-catenin paracrine signaling disrupts fatty acid oxidation and indoleamine 2,3-dioxygenase-1 activity, subsequently leading to the generation of regulatory T cells, immunotolerance, and weakened immunotherapy response [131]. These PPARγ activities create a “friendly” TME for cancer survival, which also coincides with the functional trajectory of macrophage PPARβ/δ [132,133].

Nonetheless, some findings support counterarguments. For example, Cheng et al. (2016) [134] identified macrophage PPARγ as a key tumor suppressor and TAM modulator by abolishing Gpr132 expression. Van Ginderachter et al. (2006) [135] agreed that PPARγ was highly expressed in TAMs, but further stimulation with synthetic and natural ligands could sabotage TAM-induced cytotoxic T lymphocyte suppression to confer an anti-tumor effect. The overexpression of PPARγ in macrophages promotes the upregulation of *PTEN*, which is encapsulated in exosomes. The uptake of these macrophage-derived exosomes by adjacent cancer cells inhibits Akt, p38 MAPK, and migratory properties [136]. Many eicosanoids are also packaged in these exosomes to achieve paracrine stimulation of PPARγ and augment the inhibitory effect on tumor EMT [136].

Taken together, PPARγ acts as a master immuno-metabolic switch in immune cells that govern their fate and tumor-supporting role. Current consensus depicts that PPARγ exhibits a pro-tumorigenic effect in immune cells by promoting alternative activation, which contradicts its anticancer properties in tumor epithelium and CAFs. On the other hand, the related information on other PPAR isotypes in this aspect is somewhat limited. Interestingly, a recent study unveiled that fatty acid-enriched cancer exosomes markedly activate PPARα in tumor-infiltrating dendritic cells, resulting in mitochondrial overdrive and impaired dendritic cell-mediated CD8^+^ cytotoxic T-cell priming [137]. These exciting findings strongly suggest an immuno-metabolic regulatory role of PPARα in the TME similar to PPARγ. Such a novel activity of PPARα warrants further investigation.

#### 3.4.3. Role of ANGPTL4 in Stromal–Epithelial Crosstalk

Growing evidence suggests a role of angiopoietin-like 4 (ANGPTL4) in cancer and stromal-epithelial communication. ANGPTL4 is a secretory protein that belongs to a family of ANGPTL proteins that share high amino acid sequence similarity with the angiopoietin (ANG) family [138,139]. Its expression is regulated by all three PPAR isotypes and PGE2, especially during major metabolic challenges such as starvation and hypoxia [139,140,141]. The native full-length ANGPTL4 can undergo proteolytic cleavage to yield C-terminal (cANGPTL4) and N-terminal (nANGPTL4) chains, each with distinct biological activities [142]. The nANGPTL4 domain is primarily responsible for lipid and glucose metabolism, while the cANGPTL4 domain is closely linked to tumorigenic activities, notably angiogenesis, anoikis resistance, and metastasis [143]. Thus, we will be focusing more on the cANGPTL4 fragment.

High expression of ANGPTL4 has been reported in ovarian, urothelial, and breast tumor biopsies, particularly in the CAAs [144,145,146]. The ANGPTL4 overexpression in CAAs is directed by IL-1β from neighboring TAMs with activated NLRC4 inflammasome and can be exacerbated by tumor hypoxia [147], resulting in cANGPTL4 aggregation in the TME. The cANGPTL4 interacts with integrins β1, β5, α5β1, VE-cadherin, and claudin-5 to induce PAK signaling and weaken cell–cell contacts [148,149]. Moreover, it also disrupts cell–ECM communication through its interaction with vitronectin and fibronectin [150]. The destabilization of cell junctions is then translated to greater intratumoral vascularization and migratory capacity of the malignant cells [151,152,153].

By manipulating redox homeostasis and activating several pro-survival mechanisms such as FAK/Src, PI3K/Akt, Erk signaling, ANGPTL4 markedly sharpens the resilience of tumor cells and confers anoikis resistance [154,155,156]. Our latest report showed that exogenous ANGPTL4 activates macrophages and induces hypercytokinemia via PI3K/Akt-mediated complement component 5a (C5a) activation [157]. This finding indicates a modifying role of ANGPTL4 in TAM functionality and paracrine signaling in the TME. Thus, ANGPTL4 may act as a powerful autocrine and paracrine signaling effector of PPARs that can shape a supportive environment for cancer progression. Further investigations on the therapeutic feasibility of targeting ANGPTL4 are warranted.

### 3.5. Stromal PPARγ Modulates Tumor Metastasis

Only a handful of studies have investigated stromal PPAR activities on metastasis, and the results are conflicting. In myeloid-derived suppressor cells (MDSCs), deficiency of lysosomal acid lipase (*lal^−/−^*) impaired the production of PPARγ ligands, which led to reduced PPARγ activity, ROS accumulation, and mTOR-mediated tumor metastasis [158]. Following intravenous injection of B16 melanoma cells, increased lung metastases were observed in mice with myeloid-specific PPARγ knockout, further reinforcing the role of MDSCs’ PPARγ in metastasis. Contradictorily, a PPARγ agonist, pioglitazone, has been shown to promote alternative activation of macrophages in the TME [159]. These pro-tumorigenic myeloid cells can synthesize TGFβ1 to promote EMT of surrounding tumor cells [160]. Although the true role of stromal PPARγ in metastasis remains debatable, a recent study showed that astrocytes liberate polyunsaturated fatty acids, which are PPARγ agonists, to promote the extravasation of circulating cancer cells into the brain while PPARγ antagonists can reduce brain metastatic burden in vivo [161]. Astrocyte–cancer cell communication is also mediated by TGF-β2 and ANGPTL4, the latter of which is an effector of PPARs [162]. Hence, PPARγ may serve as a nutritional cue to provoke the invasion of metastatic cells into a nutrient-rich environment. The results also argue for the potential use of PPARγ blockade to treat brain metastasis.

## 4. Knowledge Gaps and Prospects of Targeting PPARs in Tumor Stroma

### 4.1. Pressing Questions in Current PPAR Cancer Research Paradigm

Our understanding of the role of PPARs in cancer and the TME has expanded exponentially in the past decade. As the master switch of metabolism, PPARs and their actions are deeply rooted in key tumor-supporting cells in the TME, namely CAFs, CAAs, endothelial cells, and immune cells. However, the outcomes of PPAR manipulation are not always consistent. Disagreements and even conflicting experimental results between different stromal cells are not unusual [63,120,125]. The high context dependency remains a puzzle and, to date, no hypothesis can substantially address the variations.

To explain the disparate findings, Youssef and Badr (2011) [163] put forward three postulations: (i) off-target effects of PPAR ligands, (ii) diverse pharmacokinetic properties of PPAR agonists, and (iii) cancer stage-dependent effect, of which the first two focus on the intrinsic characteristics of the synthetic PPAR ligands while the last one is linked to the biological context of the TME. Undeniably, synthetic ligands that are supposed to target the same PPAR isotype do not always have comparable efficacy, off-targets, turnover rate and toxicities [164]. Hence, PPAR-independent activities on the carcinogenesis caused by the non-specificity of the PPAR modulators cannot be eliminated. However, many functional studies of PPAR in the TME were also reinforced by results from genetically knockout models [85,111,127,128]. Therefore, the pharmacodynamic and pharmacokinetic variations of synthetic PPAR ligands may not fully account for the observed discrepancies.

We believe that the controversial roles of PPARs in carcinogenesis should also have underlying biological rationales. One overlooked aspect is the crosstalk between PPARs and other nuclear receptors in different cancer types and stages. Classically, all PPAR isotypes form heterodimers with RXR to coordinately modulate their target genes [165]. Nonetheless, PPARs can cooperate with other nuclear receptors such as glucocorticoid receptors, estrogen-related receptors, and photoreceptor-specific nuclear receptors to form atypical heterodimers transiently [166,167]. These atypical heterodimers may regulate the expression of different sets of genes from those of the classical heterodimers, leading to diverse cell fate and behaviors [167]. The fact that the atypical heterodimers are not commonly detected suggests that the protein–protein interaction is labile and can only be stabilized with a unique combination of physiological cues, microenvironment, bioavailability of the co-factors and cognate ligands. The striking intra- and inter-heterogeneity of the TME, coupled with numerous unorthodox cellular activities, may be adequate to accomplish all sorts of stringent biological environments necessary for the stabilization of different PPAR-dependent atypical heterodimers. Such a flexible and highly amendable transcriptional regulatory mechanism mediated by PPAR–nuclear receptor collaboration may answer some of the disparities observed in PPAR cancer research. Nonetheless, the concept remains highly speculative. While it may explain the context-dependency of the PPAR-related carcinogenic roles, the real challenge is to experimentally capture the transient heterodimers and dissect their endogenous biological activities [167]. Nevertheless, the ability to rewire the non-canonical nuclear receptor crosstalk in the TME may offer a new therapeutic strategy in oncology considering the marked druggability of most nuclear receptors.

Another pitfall in PPAR cancer research is that current drug development and research attention highly skew towards PPARα and PPARγ. Our knowledge on PPARβ/δ and choices of PPARβ/δ-targeting drugs is comparatively limited. Yet, unlike PPARα and PPARγ, PPARβ/δ, which is ubiquitously expressed in almost all tissues, displays an apparent pro-tumor activity. Hence, potent PPARβ/δ antagonists may offer some fruitful outcomes in cancer treatment.

### 4.2. Future Prospects and Strategies to Target Stromal PPARs for Precision Oncology

**Figure 6:**
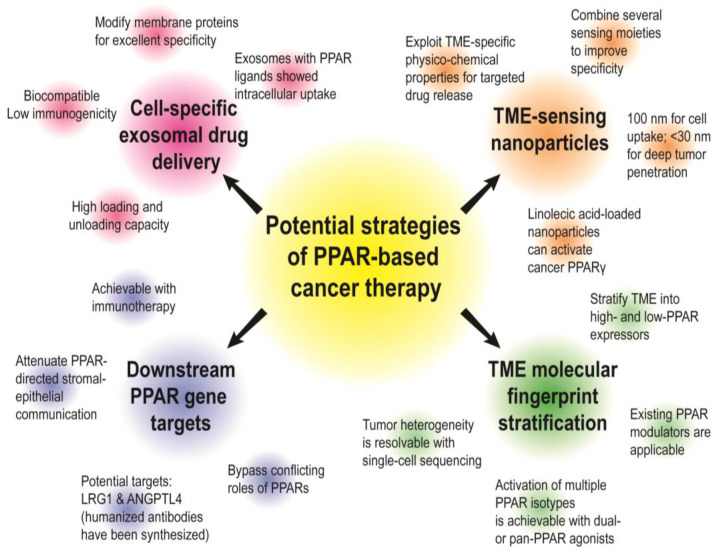
Possible strategies to target stromal PPARs for precision oncology. In this review, we propose four strategies to achieve PPAR-based precision oncology, including (i) cell-specific exosomes, (ii) TME-sensing nanoparticles, (iii) targeting pro-tumorigenic PPAR gene targets with immunotherapy, and (iv) stratification of PPAR-related TME molecular fingerprints. The features of each strategy are summarized in the figure.

Owing to the controversial roles of PPAR in the TME, the success of PPAR-based anticancer treatment potentially relies on innovative strategies for cell-type-specific drug delivery or TME-triggered drug release systems [168] (Figure 6). In this context, exosomes are excellent candidates to be developed into precise drug carriers. They are naturally occurring, hence exhibiting remarkable biocompatibility and bioavailability with limited immunogenicity [169]. Furthermore, by modifying the membrane protein compositions, exosomes have shown excellent specificity to recognize a selected protein [170] or cell type [171]. They also possess high drug loading and unloading capacity [172]. The phospholipid bilayer effectively contains the cargo from systemic drug release [169]. These striking features of exosomes allow them to be used as a targeted drug delivery system for pharmacotherapy. In fact, exosomes loaded with natural PPAR ligands such as fatty acids and eicosanoids are easily internalized, leading to high intracellular retention of the biomolecules [136,137,173]. Therefore, by carefully selecting the membrane protein targets of the exosomes, it may become possible to achieve stromal-specific administration of PPAR agonists or antagonists.

Recent advancements in TME-responsive drug release with nanoparticles are remarkable [174]. Unlike exosomes, which depend on membrane proteins to promote targeted delivery, the TME-sensing moieties of nanoparticles are usually based on physico-chemical alterations of the TME such as acidic pH, redox imbalance, high ATP and the enrichment of extracellular enzymes (MMPs and β-galactosidases) or paracrine signals (PDL-1) [174]. Nanoparticles of about 100 nm in diameter demonstrate desirable cellular uptake, and for deep tumor penetration, nanoparticles smaller than 30 nm should be used [175]. Superparamagnetic iron oxide nanoparticles functionalized with conjugated linoleic acid have been shown to increase PPARγ activity, subsequently triggering necrotic cell death in cancer cells [176]. Clearly, the nanoparticle-mediated delivery of PPAR ligands is a viable anticancer strategy. By incorporating different combinations of TME-sensing moieties within a single carrier, we can fabricate multi-sensing nanocarriers which execute drug release only when a specific cell type or set of physiological conditions is met [174]. However, singularly targeting one stromal cell type is not sufficient. For example, fibroblast activation protein-α (FAP) is a transmembrane prolyl endopeptidase highly expressed in CAFs [177]. Sibrotuzumab, a FAP-neutralizing antibody, failed to achieve even one complete or partial remission in a phase II trial involving 25 patients with metastatic colon cancer [178]. Another phase II trial with talabostat, a small molecule inhibitor of FAP, also yielded disappointing patient outcomes [179].

We can further restrict stromal–epithelial crosstalk by targeting downstream paracrine signals with immunotherapy. With careful selection of the drug candidates, immunotherapy can effectively shut down critical communication conduits between cancer cells and stromal cells. We have previously examined the feasibility of a nuclear receptor-based partitional strategy by targeting CAFs of skin squamous carcinoma [62]. The treatment disrupted stromal–epithelial communication, reduced xenograft tumor growth, and prevented the recurrence of chemoresistant cancer. Mounting evidence also supports the exploitation of molecular targets downstream to PPARs. In this review, we highlight LRG1 and ANGPTL4, which are key mediators of metastasis and EMT. Immunotherapy targeting these two molecules may effectively shut down PPAR-directed communication between tumor epithelium and stroma. Importantly, humanized neutralizing antibodies targeting these proteins are readily available [154,180].

Another step towards effective PPAR-mediated therapy is by stratifying cancer patients and predicting their susceptibility to PPAR drugs based on tumor genetic and transcriptomic profiles. Cancer patients may be stratified into low- and high-expressors of a specific PPAR isotype either in the stromal cells or cancer cells. New generation dual PPARs agonists may be administered to maximize their anticancer effect on the stromal and cancer cells. The heterogeneity of tumors is a technical challenge, which can be addressed using single-cell sequencing. Identifying molecular fingerprints between stromal and tumor cells in the actual TME will also be critical for a highly precise stratification strategy that enables existing PPAR-targeting drugs to be put to clinical use immediately. Additionally, the emergence of next-generation PPAR modulators [6], such as the selective PPARα modulator, pemafibrate, and dual- and pan-PPAR agonists such as saroglitazar, elafibranor, lanifibranor, and chiglitazar, brings about new prospects to PPAR cancer research. We anticipate that the investigation of newer PPAR modulators and their anticancer effect in the TME will gain momentum in the years to come.

## 5. Conclusions

Despite the impacts of PPAR activities on different aspects of tumor stromal–epithelial communication and tumor progression, it is not possible to ascribe either an exclusive pro- or anti-tumorigenic role for different PPAR isotypes. This is due to controversies and/or PPAR dual activities on cancer types and different stromal cell types. Likewise, conventional agonists and antagonists which target PPARs systemically may be counter-productive, considering their differential role in cancer and stromal cells, as reflected by the outcome of existing clinical trials. Targeting PPARs in the TME still holds a great deal of untapped potential. However, there is an urgent need to devise highly specific and precise strategies to target the nuclear receptors in different stromal cells to accomplish precision medicine in cancer therapy.
